# Weak coupling between energetic status and the timing of reproduction in an Arctic ungulate

**DOI:** 10.1038/s41598-024-56550-z

**Published:** 2024-03-16

**Authors:** N. J. C. Tyler, E. Post, D. G. Hazlerigg

**Affiliations:** 1 Centre for Saami Studies, UiT The Arctic University of Norway, N-9037 Tromsø, Norway; 2 Department of Agricultural Sciences, Lincoln University, Christchurch, New Zealand; 3 Department of Wildlife, Fish, and Conservation Biology, UC Davis, Davis, CA USA; 4 Department of Arctic and Marine Biology, UiT The Arctic University of Norway, N-9037 Tromsø, Norway

**Keywords:** Ecology, Physiology

## Abstract

Bioenergetic constraints are the ultimate determinant of the timing of reproduction, and seasonal breeding is consequently a widely observed trait. Consistent with this, attention has focused on plasticity in reproductive phenology conceptualized as a response to concomitant advances in the phenology of the environmental energy supply caused by climate change. Few studies, however, have directly compared timing of reproduction with energetic status in free-living wild animals. Here we demonstrate that neither body mass nor adiposity are strong proximate predictors of date of conception in wild reindeer (*Rangifer tarandus*). Weak coupling between energetic status and the phenology of reproduction accounts for the increasing discrepancy between the phenology of forage (energy supply) and the phenology of reproduction (energy demand) observed across the last 2–4 decades in two populations of this species. The results emphasise that phenological plasticity is not a passive response to changes in energy supply but derives from the way in which environmental factors interact with the core control mechanisms that govern timing. Central in this respect is integration, within the rheostatic centres of the hypothalamus, of information on nutritional status with the circannual life-history calendar.

*‘… in all the higher animals sexual periodicity, while conditioned by the environment, is regulated in its successive phases by the combined integrative action of the nervous and endocrine systems*^1^.

‘*In general terms, whenever a consistent seasonal change is observed in any organism, it is most likely to be regulated by an endogenous timing mechanism, rather than by a passive response to the environment*^2^.

Bioenergetic constraints are the ultimate determinant of how animals organise the timing of reproduction across the annual cycle, and seasonal breeding is consequently a widely observed trait. Consistent with this, plasticity in reproductive phenology, conceptualized as a response to changes in the phenology of the environmental energy supply caused by climate change, has emerged as a topic of major interest^3^. The fitness effects of phenological shifts in the nutritional environment depend, first, on the extent to which individual organisms are able to adjust the timing of their breeding phase to ambient conditions (‘phenological plasticity’) and second, on the scope for adaptation to novel temporal regimes through selection of heritable variation in plasticity (‘micro-evolution’)^4,5^. Recent studies confirm that individual plasticity is subject to selection under climate change^6^ while historical selection may reduce individual variation—and hence limit the scope for evolution of phenological plasticity^7–11^.

As an individual trait, phenological plasticity derives from the way in which environmental factors interact with the core neuroendocrine control mechanisms that govern the timing of reproduction. These integrate information on nutritional (primarily energetic) status in relation to growth, development and the circannual life-history calendar. Phenological timing is thus not a passive response to variation in the environmental supply of energy: it is governed by internal programmes which allocate energy to reproduction according to life-history requirements. Metabolic programming accounts for the otherwise inexplicable diversity of the ecological relationships that characterise species’ phenology, such as the way in which breeding is associated with increasing energetic status in some species (e.g., spring breeding rodents)^12^ but decreasing energetic status in others (e.g., autumn breeding ungulates)^13^.

Most of what is known about the relationship between energetic status, circannual calendar function and the timing of reproduction in mammals derives from experimental studies of select species (chiefly hamsters and domestic sheep)^14^. We are unaware of any field study of reproductive phenology that has examined the influence of energetic status, measured directly in terms of adipocity, on timing of breeding at an individual level. Most invoke either meteorological or associated environmental parameters (e.g., ambient temperature, rainfall, date of green-up) or population density as proxies for the provision of, or demand for, environmental energy, but which provide no information on individual differences in energy state, or indices like individual body mass with attendant uncertainties about resultant changes within the animal^15–19^. Analyses of taxonomic variation in phenological sensitivity to climate change, moreover, have commonly been performed without integration of the temporal programmes that govern the responses they aim to explain^20–24^.

Bronson^12,25^ proposed that the interaction between energetic status and temporal programming which defines phenological plasticity was a function of species’ life-history including, in particular, the length of the female reproductive cycle. In some mammals, gestation is sufficiently short for activation of the reproductive axis, courtship and mating to occur within the same season as parturition and lactation; in others, long gestation dictates that mating and parturition necessarily occur in different seasons. Bronson suggested that species with a short temporal separation between mating and parturition would potentially find environmental energy supply a sufficiently reliable indicator of breeding opportunity for them to adopt a flexible breeding strategy and hence to respond opportunistically to variation in conditions. Lengthy separation between the calendar phases of conception and parturition, by contrast, would weaken the link between the environmental energy supply at mating and lactation, hence favouring what Bronson termed “the predictor option”. In such cases, he proposed, reproductive timing was likely to depend largely on circannual mechanisms entrained by photoperiod. The annual cycle of photoperiod is an invariant entraining signal, and species reliant on endogenous timing would therefore be expected to show only a small degree of environmental plasticity in reproductive phenology.

In the present study we used individual measurements of energy (fat) reserves to examine the influence of energetic status at mating on the phenology of reproduction in reindeer/caribou (*Rangifer tarandus*, hereafter ‘*Rangifer*’). *Rangifer* is an interesting model in which to examine the link between energetics and reproductive timing for two reasons. First, the characteristically high degree of reproductive synchrony which this species displays seems little affected by energetic state. More than 95% of conceptions occur within 15 days in populations characterised by high and low adiposity in females at breeding (100% and zero prevalence of fat reserves, respectively; Table 1). Second, *Rangifer* display low phenological plasticity: the rates of change in the timing of reproduction associated with directional climate change observed in this species are among the lowest recorded in large ungulates (≤ 0.15 days·year^−1^; Table 2, Fig. 1; see also^36–38^). These population level responses suggest that metabolic modulation of reproductive timing is subordinated in *Rangifer* to a tightly constrained circannual programme such that individual variation in energetic state at breeding has little influence on the timing of conception. Here we extend this framework to the individual level and find negligible effect of either body mass or adipocity on the date of conception (DoC) in Svalbard reindeer (*R. t. platyrhynchus*). This result accounts for the low level of environmental sensitivity in reproductive phenology evident in *Rangifer* and emphasises the importance of circannual metabolic programming in determining species differences in plasticity among mammals.

**Table 1:** Contrasting population level associations between energetic state and synchrony of reproduction in adult females of three sub-species of reindeer/caribou *Rangifer tarandus*.

Sub-species	Location	Synchrony	Subcutaneous fat		Sources
		Days (*n*)	Prevalence % (*n*)	Mean depth mm (s.d., *n*)	
*tarandus*	South Georgia	9^a^ (26)	0 (22)	n/a	^26^
*groenlandicus*	Canada	11^b^ (48)	74 (74)	15 (^c^, 95)	^27,28^
*platyrhynchus*	Svalbard	15.3^b^ {14} (57)	100 (56)	45 (7.1, 56)	This study

Energetic state: prevalence and depth of subcutaneous fat (the largest deposit in these animals^28,60^) measured at or soon after breeding. Synchrony: duration of the period in which > 95% of conceptions occur. ^a^ > 95% of conceptions. ^b^ All conceptions. ^c^ No s.d. reported but inspection of the data yielded s.d. = 10.6. n/a Not applicable. The value in braces was determined excluding one outlier (specimen 12/1990; Fig. 3).

**Table 2:** Plasticity in reproductive timing in different species of large ungulate.

(Sub-)species		Phenological parameter	Mean rate of change (days·year^−1^)	Duration of time series (years)	Environmental correlate	Study
Roe deer	*Capreolus capreolus*	Birth	0	27	Spring temperature	^29^
Reindeer	*Rangifer tarandus platyrhynchus*	Birth	− 0.05 *NS*	37	Date of melt	This study
Caribou	*R. t. groenlandicus*	Birth	− 0.11	33	Date of green-up	^30^
Reindeer	*R. t. tarandus*	Birth	− 0.15	45	Late winter temperature	^31^
Red deer	*Cervus elaphus*	Oestrous	− 0.26	28	Seasonal growing degree days	^17^
Red deer	*Cervus elaphus*	Birth	− 0.42	28	Seasonal growing degree days	^17^
Big horn sheep	*Ovis canadensis*	Birth	− 0.58	26	Autumn precipitation	^9^
Red deer	*Cervus elaphus*	Conception	− 1.2	12	Population density, spring real bioclimatic index, body mass	^32^
Chamois	*Rupicapra pyrenaica*	Birth	− 1.3 to − 3.9	22	Onset of autumn and of spring	^19^

Rates of advance (days·year^−1^; negative values indicate an advance in date) in population mean dates of oestrous, conception or birth associated with warming or environmental correlates of warming.

**Figure 1:**
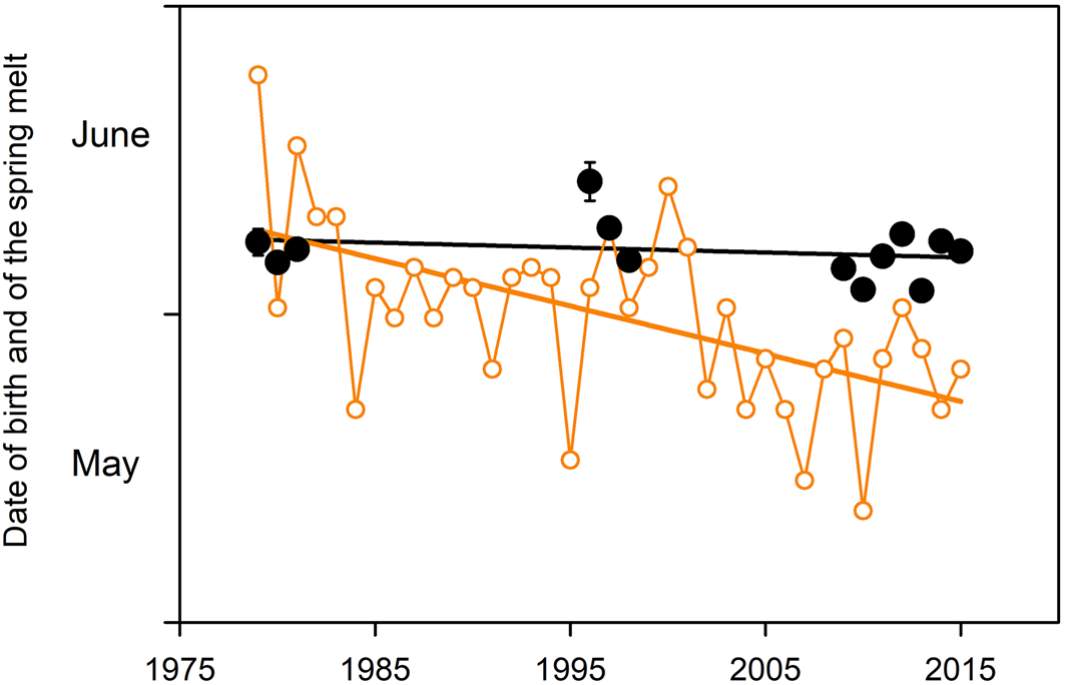
Reproductive phenology and trends in the weather in Svalbard in the study area (Supplementary Fig. S1). Annual mean date of calving in Svalbard reindeer (black circles; s.e.m. bars fall within the symbols) and annual date of the onset of the melt in spring (orange circles) in the study area. The melt is an index of the start of the annual season of plant growth and hence of the phenology of forage for reindeer^33^. The melt has advanced 17 days across 36 years while the timing of calving has not changed significantly. Consequently, calving falls increasingly later relative to the melt across the time series with the result that the peak of lactation is in turn increasingly likely to fall after the first flush of forage in summer. The discrepancy between the timing of the melt and calving among reindeer in Svalbard closely matches a discrepancy between the timing of the green-up and calving which has developed since the late 1990s among caribou in West Greenland^30^. The fitted lines are linear regressions: date of birth (± 1 s.e.m.) = − 0.049 (0.059) year + 255.330 (118.657), *r*^*2*^ = 0.058, *P* > 0.4 *NS*; date of melt (± 1 s.e.m.) = − 0.468 (0.113) year + 1086.328 (226.349), *r*^*2*^ = 0.328, *P* < 0.001. Reindeer data ^34,35^. Weather data: Norwegian Meteorological Institute.

## Results

### Timing and synchrony of conception

**Figure 2:**
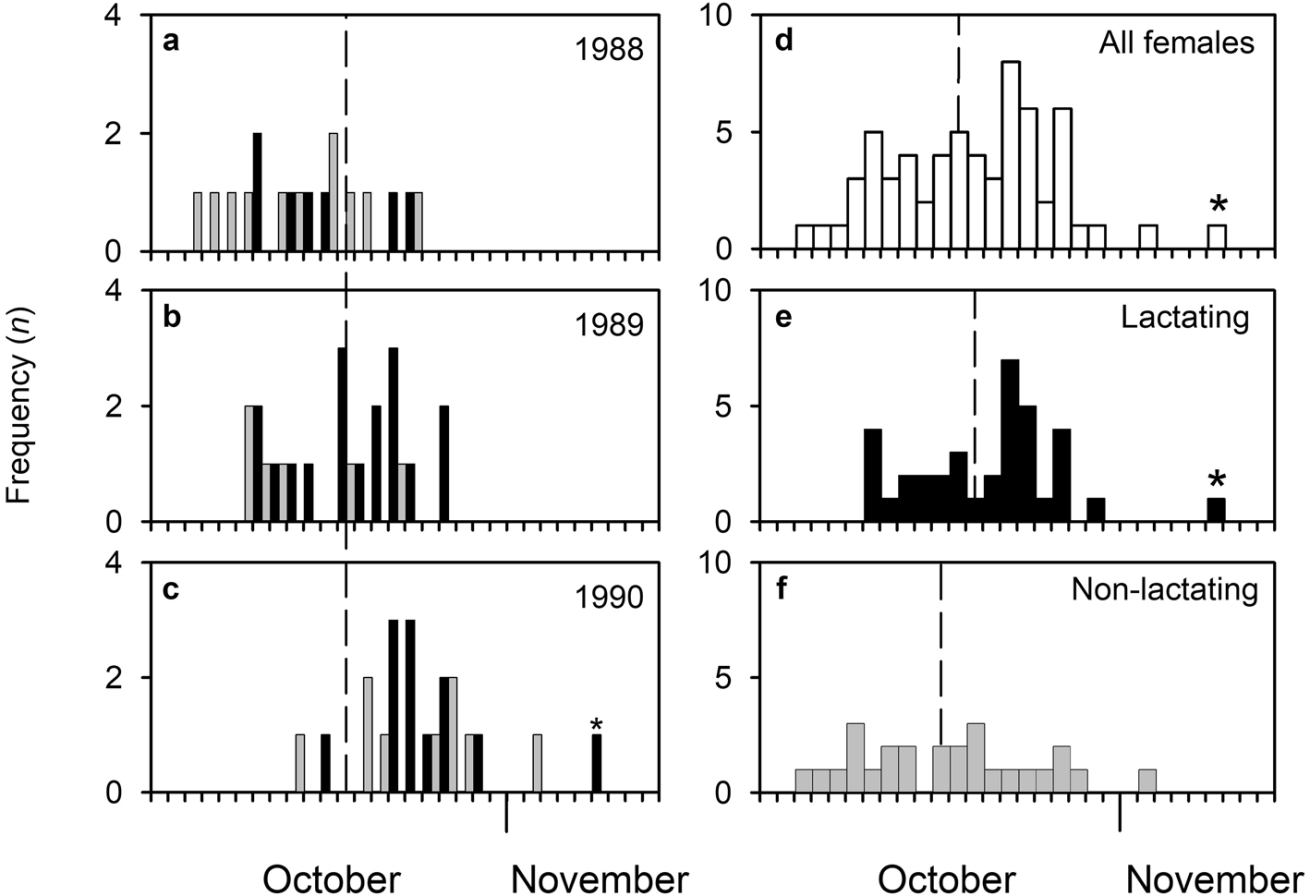
Temporal distribution of date of conception (DoC) in Svalbard reindeer aged 1–11 years (age distribution of the sample: Supplementary Fig. S4). Median DoC (dashed lines): (**a**–**c**) lactating females (black bars) and non-lactating females (grey bars) by year and (**d**) combined sample (all females, all years) 22nd October (s.d. 4.6 days); (**e**) lactating females (all years) 23rd October (s.d. 4.1 days); (**f**) non-lactating females (all years) 21st October (s.d. 5.4 days). DoC was significantly associated with reproductive status singly and through interactions with age, and depth of subcutaneous fat over the rump (RFD, an index of total dissectible body fat; see Methods) and carcass mass, respectively, and with age singly and through interactions with reproductive status, RFD and carcass mass, respectively (Table 4). Black star outlier (specimen 12/1990) referred to in Table 3, Fig. 3 and Supplementary Table S1.

DoC was normally distributed in all three years (1988, *JB* = 0.45, *P* > 0.7 *NS*; 1989, *JB* = 1.09, *P* > 0.05 *NS*; 1990 *JB* = 3.90, *P* > 0.1 *NS*; Fig. 2). The overall median DoC was 22nd October (s.d. 4.6 days). Annual median DoC ranged from 18th to 26th October (*P* < 0.001; Table 3). All conceptions occurred within 13–19 days annually (mean = 15.3 days) and between 65.2 and 81.0% of conceptions occurred within 4 days of the annual median date (mean 72.6%; Table 3).

**Table 3:** Temporal pattern of conception and energetic state at breeding in Svalbard reindeer.

	Date of collection	Date of conception					Total dissectible fat (kg)			*n*
		Median	Earliest	Latest	Range (days)	% within 4 days of the median date	Median	Min	Max	18
1988	21–28 Nov.	19 Oct.	13 Oct.	26 Oct.	14	72.2	14.5	6.9	18.1	18
1989	29 Nov.–14 Dec.	24 Oct.	16 Oct.	28 Oct.	13	65.2	11.0	3.7	18.6	23
1990	23 Nov.–8 Dec.	26 {25} Oct.	19 Oct.	6 {2} Nov.	19 {15}	81.0 {85.0}	11.5 {11.3}	7.7	21.2	21
		***								
All years	21 Nov.–14 Dec.	22 Oct.	13 Oct.	6 {2} Nov.	15.3^a^ {14}	72.6 {73.8}	12.6	3.7	21.2	62

**Figure 3:**
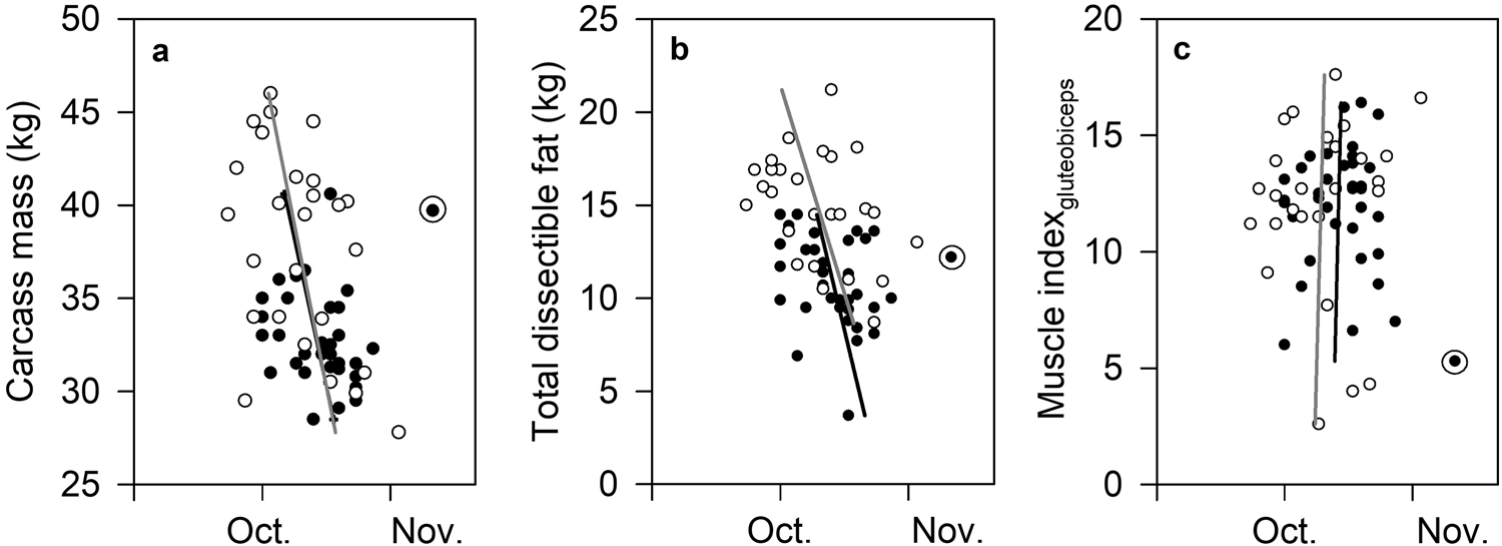
Associations between three indices of energetic state at breeding and the date of conception (DoC) in Svalbard reindeer aged 1–11 years (age distribution of the sample: Supplementary Fig. S4). (**a**) carcass mass (CM, kg); (**b**) total dissectible fat (TDF, kg; estimated from the depth of subcutaneous fat over the rump (RFD, mm) with which it was strongly positively related; see Methods); (**c**) Muscle Index_*gluteobiceps*_ (MI_g_; see Methods). Data are distinguished by reproductive state: lactating females (black circles, black regression lines); non-lactating (open circles, grey regression lines). By convention the axes are reversed so that the temporal variable is plotted on the abscissa. Coefficients, goodness of fit statistics and significance of linear regressions are given in Table S2. (Linear regressions for lactating females were calculated excluding one outlier (specimen 12/1990; ringed) identified on the basis of its standardized residual being > 3.5^39^.) DoC was significantly associated with reproductive status singly and through interactions with age and RFD, respectively, and with age singly and through interactions with reproductive status, RFD and carcass mass, respectively (Table 4). Association between three other indices of energetic state and DoC are illustrated in Supplementary Fig. S3.

Date of collection of specimens (first and last date), estimated dates of conception and total amount of dissectible fat (kg) in carcasses of female Svalbard reindeer aged 1–11 years (age distribution of the sample: Supplementary Fig. S4). Date of conception was estimated from gestational age based on embryonic length (see Methods). ^a^ mean. Values in braces were determined excluding one outlier (specimen 12/1990; Fig. 3). *** *P* < 0.001. Data for a further five indices of energetic state are given in Supplementary Table S1.

### Effect of age, energetic status and reproductive status on date of conception

Energetic status at breeding differed greatly among individuals: total dissectible fat in the reindeer, for instance, varied almost six-fold, from 3.7 to 21.2 kg (median 12.6 kg, *n* = 61). Visual inspection of the data indicated in each case that indices of energetic status had only a minor influence on the timing of conception (Fig. 3, Supplementary Fig. S3). Modelling confirmed this. In neither family of models (i.e., those with rump fat depth (RFD) or carcass mass, respectively, included as a predictor) was energetic status a significant individual predictor of DoC (Table 4: Models 1, 3, 5, 7). However, the best-performing model (based on minimization of the Bayesian Information Criterion score) in the family of models that included RFD as a predictor identified a significant interaction between age and RFD (Table 4, Model 2). Similarly, the best-performing model in the family that included carcass mass as a predictor identified a significant interaction between age and carcass mass (Table 4, Model 6). In both cases, the coefficient estimates for these interaction terms indicated earlier dates of conception in older and fatter or heavier animals, though this effect was minor in both models (Model 2: b (± 1 s.e.m.) = -0.01 (0.004); Model 6: b (± 1 s.e.m.) = − 0.01 (0.005)). Slightly less well-performing models in both families revealed significant interactions between reproductive status and age, and between reproductive status and energetic status, on DoC. In the former, non-lactating females (but not lactating females) conceived earlier at older ages (Table 4, Models 3 and 7). In the latter, fatter non-lactating females (but not lactating females) conceived earlier (Table 4, Model 4). No model in either family revealed a significant effect of muscle protein on DoC (Table 4).

**Table 4:**
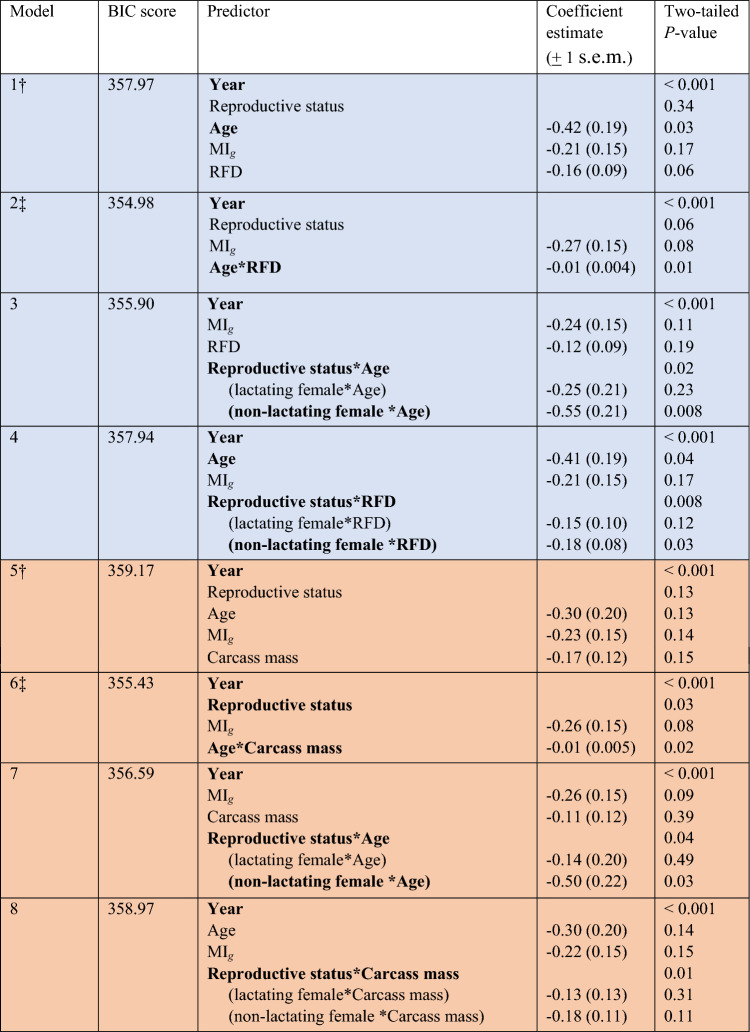
Summary statistics of generalized linear model (GLM) analyses of date of conception in Svalbard reindeer.

Models comprise two families: those including the depth of subcutaneous fat over the rump (RFD, mm) as a predictor (blue shading) and those using carcass mass (kg) as a predictor (orange shading). In each family of models, the baseline model (†) using only individual predictors is reported first, followed by the best overall model (‡) within each family (allowing for interactions between predictors) as determined by lowest Bayesian Information Criterion (BIC) score. Bold terms are significant (*P* < 0.05). Coefficient estimates are calculated only for continuous predictors. MI_*g*_ Muscle Index_*gluteobiceps*_*.*

## Discussion

This study demonstrated that energetic status at mating has little effect on the timing of reproduction in female Svalbard reindeer. Realisation of high reproductive synchrony despite very large individual variation in energetic state (Table 3, Fig. 3, Supplementary Table S1 and Fig. S3) indicates that the influence of metabolic feedback on the reproductive axis is tightly constrained in this sub-species. The resulting low level of environmental sensitivity, manifest as low annual rates of advance in the timing of births in Svalbard reindeer and other *Rangifer* (Table 2), accounts for the increasing discrepancy, under climate change, between the phenology of forage (energy supply) and the phenology of reproduction (energy demand) observed across the last 2–4 decades in West Greenland caribou (*R. t. groenlandicus*)^30^ and Svalbard reindeer (Fig. 1). Our results emphasise that reproductive timing is not a passive response to environmental variation but derives instead from integration, within the rheostatic centres of the hypothalamus, of multiple inputs, including nutritional and lactational status and age (Table 4), with the circannual life-history calendar.

Our estimates of the temporal pattern of conception in Svalbard reindeer are closely similar to those reported for other sub-species of *Rangifer*. The median DoC of 22nd October (Table 3) compares with an estimate of 24th October in barren-ground caribou (also *R. t. groenlandicus*)^27^. The level of synchrony in Svalbard reindeer (all conceptions within 15.3 days annually) compares with estimates of 11 and 9 days in *R. t. groenlandicus* and European tundra reindeer *R. t. tarandus*, respectively (Table 1). On average 73% of conceptions in Svalbard reindeer occurred within four days of the annual mean DoC (Table 3) compared to 71% within 2 days in *R. t. groenlandicus*^27^. The similarity of the temporal pattern between sub-species indicates both the reliability of the retrospective method for determining the date of conception and that a low level of plasticity in reproductive timing is conserved across the genus.

Typical for mammals, return to oestrous was influenced by reproductive status. Lactating females conceived later than non-lactating females but, consistent with the high level of synchrony and the low level of plasticity in Svalbard reindeer, the difference between the median date of conception in the two classes was small (2.2 days; Fig. 2) compared to corresponding values in temperate zone species (7–12 days in red deer (*Cervus elaphus*) and sika deer (*C. nippon*)^40–42^.

Reproductive timing correlates with indices of energetic state, such as body mass and population density, in phenotypically plastic species of cervids (e.g., red deer, moose (*Alces alces*) and white-tailed deer (*Odocoileus virginianus*)^15,16,18,32,42–44^. The timing mechanisms of these species evidently respond to metabolic feedback and the absence of plasticity in species such as roe deer (*Capreolus capreolus*; Table 2) has, by extension, implicitly been attributed to insensitivity of control mechanisms to the same stimulus^29^. Differences in the sensitivity of control systems to metabolic feedback are not, however, sufficient explanation for species’ variation in plasticity. Such a view omits the modulation of stimulatory metabolic and other input by the regulatory centres that govern the temporal response^12,14^. The low sensitivity of the timing of reproduction to energetic status in Svalbard reindeer aligns instead with the concept of sensitivity to energetic status being constrained within a temporal window determined by circannual programming.

**Figure 4:**
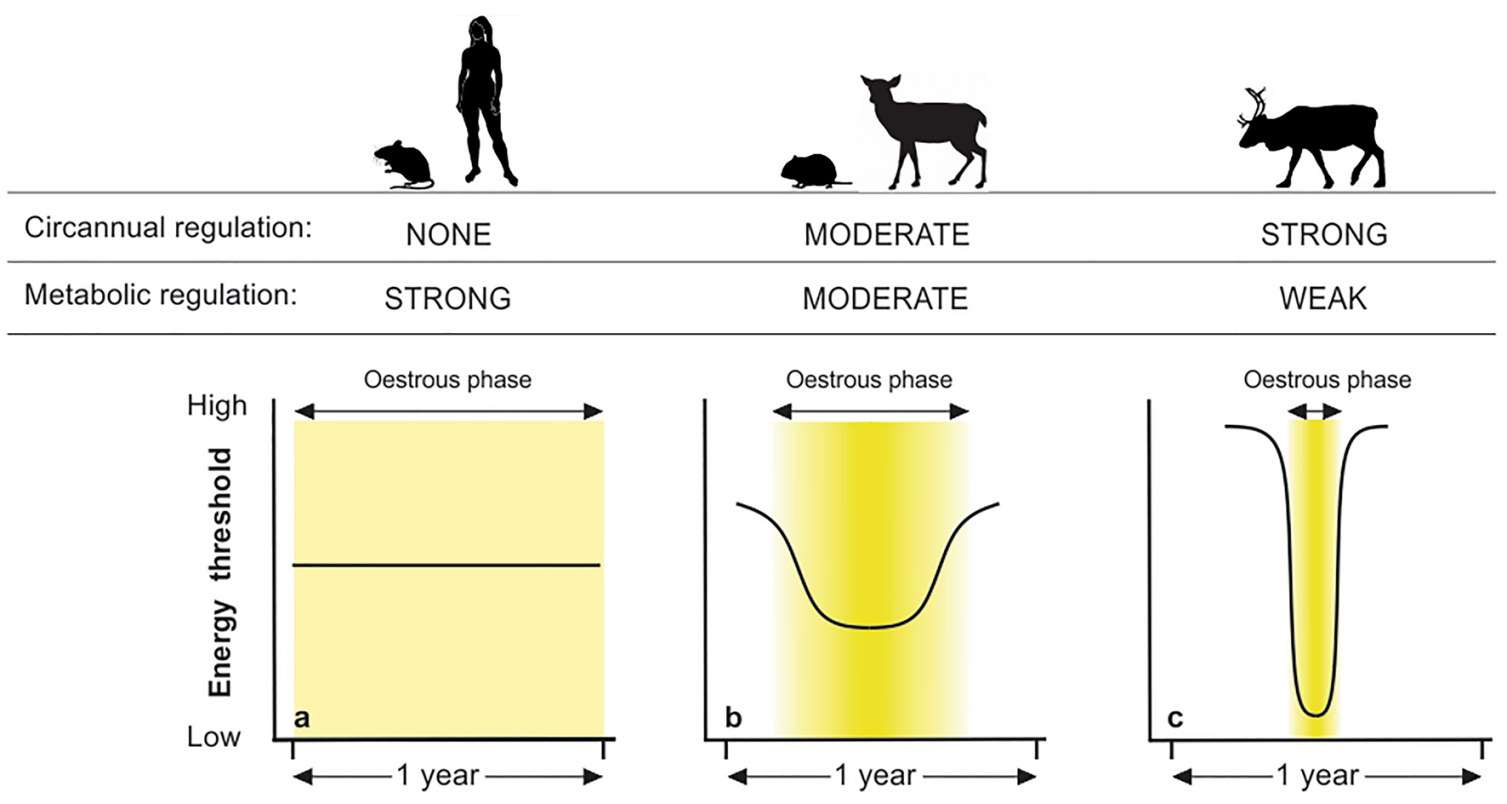
Model of constant versus circannually programmed energy thresholds for reproduction. (**a**) Opportunistic breeders (e.g., house mouse^48^, humans^46,49^). The energy threshold for the activation of the reproductive axis (horizontal solid line) in opportunistic breeders is envisaged as intermediate and constant. Energetic state can readily exceed this threshold and individuals which have attained an appropriate level of post-natal development may therefore potentially breed at any time of year. (**b**) Moderate circannual control of the timing of breeding (e.g., microtine rodents^12,50^, equatorial and temperate zone deer^2,42^, sheep^51,52^). The activation threshold is envisaged having an approximately sinusoidal form. The energetic state of the animals is consequently unlikely to exceed the threshold outside the low phase of the cycle which in turn restricts reproductive activation to the time of year corresponding with this phase. The slow rate of change of the threshold, however, means that both the onset and the offset of oestrous phase are weakly defined which consequently leaves scope for metabolic modulation of the timing of reproduction at either stage. Species with these characteristics show plastic seasonal timing. (**c**) Strong circannual control of the timing of breeding (e.g., *Rangifer*[this study]). The activation threshold is envisaged having a high amplitude sinusoidal form. The normally high energy threshold entirely prevents (re)activation of the reproductive axis outside the oestrous phase of the annual cycle while the programmed low threshold within this phase is permissive such that even animals in low energetic state may breed. Strong circannual regulation, manifest by the rapid rate of change between the high and low threshold phases, reduces the scope for metabolic modulation of reproduction to a minimum. Species with these characteristics show low levels of plasticity in seasonal timing. The intensity of yellow shading in each graph indicates the population frequency of conceptions across the oestrous phase of the annual cycle.

Metabolic regulation of reproductive function in mammals is generally considered in terms of a notional energy threshold which must be exceeded for reproduction to take place^45,46^. Reproductive activation depends on the hypothalamic neuronal network which controls activity of the reproductive GnRH pulse generator, and this network assesses energetic status in relation to the notional threshold through changes in the levels of peripheral hormones (e.g., leptin, insulin) and metabolites (e.g., glucose, fatty acids)^47^. In opportunistic breeders, like house mouse (*Mus musculus*)^48^ and humans^46,49^, the notional energy threshold is considered effectively constant (Fig. 4a) and individuals may therefore breed whenever their nutritional/energetic status exceeds threshold requirements, irrespective of the calendar date at which this is achieved. This scenario leads to a high degree of energy sensitivity in the timing of reproduction.

The energy threshold in obligate seasonal breeders (Fig. 4b,c), by contrast, is envisaged as transiting between high values, that effectively shut down the reproductive axis outside the normal breeding season, and lower permissive values within it. The transition between high and low threshold phases, construed as the output of the circannual programme, sets the calendar phase of reproduction. Crucially, there is no conceptual objection in this model to interspecific variation either in the differential between the higher and lower energy threshold phases or in the slope function of the transit or in both. The model therefore offers a simple conceptual framework that encompasses the spectrum of seasonality observed in wild mammals, from seasonal opportunism (in species with a constant or low amplitude modulated threshold) to inflexible timing (in species with a step change from a high threshold, non-breeding state to a low threshold, activated state). We envisage *Rangifer* closer to the latter, with a steep slope between the high and low threshold phases (Fig. 4c). The expression in Svalbard reindeer of a low level of plasticity (Table 3, Fig. 2) despite high individual variation in energetic status (for which body mass and fat reserves were our proxies; see also^53^) thus emerges from the inherent constraint imposed on the reproductive axis during the programmed transition from high to low threshold values at the start of the breeding season (Fig. 4c).

By what mechanism might this apparent shift in energy threshold be mediated by the circannual programme? The current paradigm for seasonal activation of reproduction in mammals envisages melatonin-responsive cells in the pituitary *pars tuberalis* as modulators of tanycyte function in the basal hypothalamus, with tanycytes then acting on the neuronal network governing the metabolic energy sensitivity of the GnRH pulse generator^14^. Tanycytes have also emerged as hypothalamic sensors of bioenergetic status^54–56^, and we therefore speculate that the neuro-anatomical basis for the circannually programmed reproductive energy threshold is closely associated with tanycyte function—possibly even residing in the tanycytes themselves. Studies in the common vole (*Microtus arvalis*)^57^, a boreal rodent that displays plastic seasonal reproductive timing (Fig. 4b), are consistent with this hypothesis. Here, post-natal reproductive development is circannually modulated via in utero and post-natal photoperiod exposure, and photoperiod and temperature interact to control gene expression in tanycytes^50^. The cellular substrates of a circannual rheostat are thus beginning to be characterised.

Our analysis has two broad implications for research into seasonal reproductive plasticity in response to environmental change. First, our treatment demonstrates that a framework which places the circannual reproductive programme at the top of the regulatory hierarchy offers an intellectually satisfying means of reconciling the wide disparity in temporal shifts between even quite closely related species. Secondly, it proposes a clear focus for understanding the mechanistic origins of disparate responses to climate change. This shifts the emphasis away from purely statistical attempts to resolve the differing contributions of individual level plasticity versus population level genetic selection, which have yielded ambiguous outcomes. Productive future avenues will involve focussed analyses of epigenetic and evolutionary effects at defined genetic loci linked to circannual metabolic programming.

## Methods

### Field studies

#### Animals

Svalbard reindeer embryos were recovered from 62 females aged 1 to 11 yr. collected (licences SMS 1446/88 a512.43 AB, SMS 1536/89 a512.43 and SMS 1816/90 a512.43 AB) approximately 6 weeks after mating (median date of collection 29th November, range = 21st November to 14th December) over three years 1988–1990 in the Reindalen-Semmeldalen-Colesdalen area of Nordenskiöld Land, Svalbard (78° N, 16° E; Supplementary Fig. S1). Each animal was spotlighted and killed with a single shot to the head or the chest, using cartridges that delivered bullets with an impact energy of > 2.2 kJ at 100 m. The dead animals were taken to a field laboratory where dissection started usually within 3 h of death.

#### Reproductive tract and status

The reproductive tract was removed from each animal by severing the broad ligaments and the cranial vagina. Uteri were frozen intact and stored at − 20 °C until examination. The animals were classed as lactating or non-lactating based on palpation of the udder.

#### Body mass and physiological condition

Energetic status at mating was assessed in terms of body mass and indices of total dissectible body fat and skeletal muscle protein on the assumption that the animals were in steady state. The reindeer were unlikely to have either gained or lost energy or protein in the interval between mating and sampling owing to the expression of winter anorexia and the marginal energy requirements of simultaneous late lactation and early pregnancy, respectively^13,58^.

Hot carcass mass (CM) was measured by weighing the dressed carcass to 0.1 kg on a 100 kg × 50 g steel yard. CM is the whole animal less the entire gastrointestinal tract and its contents, the head, the pelt, the distal limbs, the uterus and its contents, the udder and all visceral organs except the kidneys and the fat adhering to them. The head was removed by cutting between the cranium and the atlas; the limbs were severed at the carpometacarpal and tarsometatarsal joints.

The total mass of dissectible fat (TDF) in each carcass was estimated from the depth of subcutaneous fat over the rump (RFD, mm), measured to 1 mm on one side in each carcass^28^, which is a reliable proxy in female Svalbard reindeer (Supplementary Fig. S2) and other *Rangifer*^59^, as:

1 $${\text{TDF}}({\text{kg}} \pm {\text{1 s}}.{\text{e}}.{\text{m}}.) = 0.{4769}\left( {0.0{824}} \right){\text{RFD}} - {8}.{84}0{9}\left( {{3}.0{711};\,\,\,\,r^{2} = 0.{77},\,P < 0.00{1}} \right)$$

Skeletal muscle protein was evaluated by dissecting out *M. gluteobiceps*, *M. semitendinosus* and the femur on one side in each animal. The muscles were weighed fresh to 1 g after which their water content was determined by removing approximately 60 g of tissue from the middle of each, which pieces were then successively weighed, dried to constant weight at 60° C in an oven, cooled in a desiccator and reweighed. The greatest length of the femur was measured to 1 mm in a bone box. Data were expressed as a muscle index value (MI)^60^:

2 $${\text{MI}} = \left( {{\text{M }}/{\text{ f}}^{{3}} } \right) \cdot {1}0^{{3}}$$

where, M = the dry mass of *M. gluteobiceps* or *M. semitendinosus* (g) and f = length of the femur (cm) cubed for uniform dimensionality.

No correction was made for the lipid content of the muscles because this is < 5% wet weight^61^.

Dry matter (DM) content of the rumen was estimated by opening the rumen along the dorsal wall, tipping out and weighing the contents (mean wet weight (WW) 11.6 kg, 2.1 s.d., *n* = 61) to 0.5 kg. The contents were mixed and a grab sample (approximately 60 g) was weighed to 0.1 g (*w*), dried to constant weight at 60° C, cooled in a desiccator and reweighed (*d*). Rumen contents DM was calculated as WW · (*d*/*w*).

### Laboratory studies

#### Embryos

Uteri were thawed at room temperature, opened from the dorsal aspect (by cutting through the dorsal wall of the cervix, the body of the uterus and each uterine horn) and the single embryo was removed from each.

#### Gestational age and date of conception

The gestational age (days) of each embryo (*n* = 62) was estimated from its length which, by convention, was measured according to its state of development^62–66^. In small specimens (≤ 20 mm), shaped like a closed letter ‘C’, we recorded the straight-line distance between the dorsal flexure to the caudal flexure (greatest length, GL). In larger specimens, shaped like an open ‘C’ or a ‘J’, we recorded the straight-line distance from the most cranial part of the head to the caudal extremity of the ischium (crown-rump length, CRL). Measurements, made to 1 mm with vernier callipers and hereafter referred to as embryo length (EL), ranged from 4 to 55 mm (median 16 mm).

**Figure 5:**
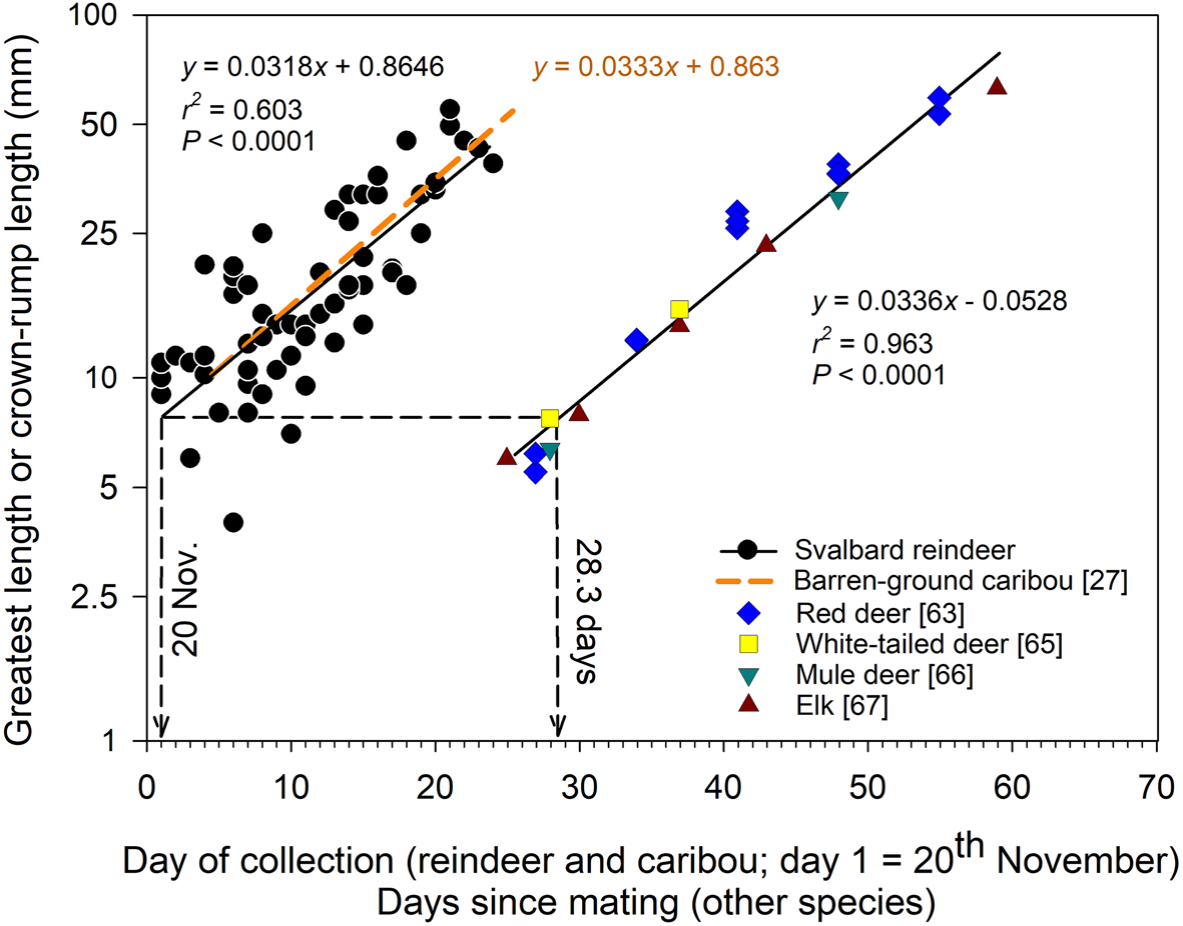
Determining the date of conception of Svalbard reindeer embryos. Greatest length or crown-rump length of embryos (EL, mm; see Methods) of barren-ground caribou (*Rangifer tarandus groenlandicus*)^30^ and Svalbard reindeer (*R. t. platyrhynchus*) by day of collection, and of red deer (*Cervus elaphus*), white-tailed deer (*Odocoileus virginianus*) mule deer (*O. hemionus*) and elk (*C. canadensis*) by known age^63,65–67^. The regression coefficients for the Svalbard reindeer and the known-age samples are not significantly different (*t* = 1.052, *df* = 79, *P* > 0.2 *NS*) and the estimate of EL in Svalbard reindeer on the first day of collection (20th November) therefore corresponds to a gestational age of 28.3 days. The gestational age of each Svalbard reindeer embryo was therefore estimated as [gestational age (days) = ((log_10_EL − 0.8646)/0.0318) + 28.3] and the date on which it was conceived was calculated by subtracting this from the calendar date of collection.

Gestational age was determined from a calibration curve constructed using data on the growth (length) of known-age embryos extracted from the literature. Log-linear rates of growth of embryos of northern species of cervids are similar from approximately 30 to at least 60 days of age (EL range 6–65 mm): regression coefficients describing the daily rate of increase in the EL of known-age embryos at this stage range from 0.031 to 0.035 (red deer^63^, white-tailed deer^65^, mule deer^66^, elk *Cervus canadensis nelson*^67^). A regression of the combined data reported in these studies took the form:

3 $$y( \pm {\text{1 s}}.{\text{e}}.{\text{m}}.) = 0.0{336 }\left( {0.00{15}} \right)x \, {-} \, 0.0{528 }\left( {0.0{617};\,\,r^{2} = 0.{963},\,P < 0.000{1}} \right)$$

where, *y* = log_10_EL (mm) and *x* = gestational age (time since mating, days; Fig. 5).

Corresponding coefficients for Svalbard reindeer embryos, where *x* = day of collection (coded as 20th November = day 1, all years combined), did not differ significantly between years. A generalized linear model of EL versus year, day of year, and year*day of year interaction revealed that the latter term was not significant (Wald Chi-Square = 1.02, *df* = 2, *P* = 0.60) while EL pooled across years was significantly positively related to day of year (DoY, 1st January = day 1; Wald Chi-Square = 58.3, *df* = 1, *P* < 0.001). The data were therefore combined in a single regression which took the form:

4 $$y( \pm {\text{1 s}}.{\text{e}}.{\text{m}}.) = 0.0{318 }\left( {0.00{33}} \right)x_{{}} + \, 0.{8646 }\left( {0.0{426};\,\,\,r^{2} = \, 0.{6}0{3},\,P < 0.000{1}} \right)$$

The coefficients of Eqs. 3 and 4 were not significantly different (*t* = 1.052, *df* = 79, *P* > 0.2 *NS*; Fig. 5). The estimate for the first day of collection (*y* = 0.8964) corresponded to a gestational age in the known-age sample of 28.3 days (Eq. 3). The gestational age of each Svalbard reindeer embryo was therefore estimated as:

5 $${\text{gestational}}\,{\text{age}}\,\left( {{\text{days}}} \right) = \left( {{{\left( {{\text{log}}_{{{1}0}} {\text{EL }}{-} \, 0.{8646}} \right)} \mathord{\left/ {\vphantom {{\left( {{\text{log}}_{{{1}0}} {\text{EL }}{-} \, 0.{8646}} \right)} {0.0{318}}}} \right. \kern-0pt} {0.0{318}}}} \right) + {28}.{3}$$

The DoC of each embryo was then determined by subtracting its gestational age from the date of collection coded as DoY. Data, verified normal using Jarque–Bera tests, were analysed as DoY and then converted back to calendar dates for non-leap years. The median DoC for each year, for lactating and for non-lactating animals was calculated by probit regression^68,69^.

#### Age of reindeer

Calving is highly synchronised in Svalbard reindeer: approximately 90% of births occur within the first 10 days of June^34,70^. The age at its last birthday (i.e., in June, 5 or 6 months prior to collection) of each reindeer in the sample was estimated to the nearest whole year by a combination of tooth eruption pattern (reindeer < 36 mo. old) and counting annulations in the cementum of decalcified sections of 1st incisor teeth (reindeer aged ≥ 36 mo.)^71–73^.

#### Data analysis

We analysed variation in DoC using two families of generalized linear models (GLMs), both with DoC as the response variable. In the first set of GLMs we included RFD as a predictor and in the second we used carcass mass as a predictor. No single model included both RFD and carcass mass as predictors because the two were significantly correlated (Pearson’s *r* = 0.66, two-tailed *P* < 0.001, *n* = 60). Both sets of GLMs also included the year of collection of the individual animal and its reproductive status as continuous and categorical factors, respectively, and MI and age of the individual animal as continuous covariates. In both sets of GLMs we used an identity link function with maximum likelihood estimation of model coefficients and Type III analysis of model effects with Wald Chi-square significance tests of individual model terms. After thus testing for the significance of individual predictor terms in baseline models of both sets of GLMs, we re-analyzed the data using the same approach but with interaction terms for all pairwise combinations of predictors in each family. This approach was intended to facilitate assessments, both within and between model families, of the significance of individual predictors and interaction terms as well as the manner in which their significance varied with increasing model complexity.

### Supplementary Information

Supplementary Information.

## Data Availability

The data analysed in this study are available from the corresponding author on request.
