# Supplementary material for: Weak coupling between energetic status and the timing of reproduction in an Arctic ungulate
Source: Sci Rep. 2024 Mar 16;14:6352. doi: 10.1038/s41598-024-56550-z (PMC11322327; doi:10.1038/s41598-024-56550-z)
Supplement: Supplementary file 1 — Supplementary Information. [file 41598_2024_56550_MOESM1_ESM.docx]

**Supplementary information for:**

Limits to phenological plasticity in mammals: why energetic status is a poor predictor of reproductive timing in an Arctic ungulate.

N. J. C. Tyler, E. S. Post, and D. G. Hazlerigg

**Table S1.** Temporal pattern of conception and energetic state at breeding in Svalbard reindeer aged 1-11 years (age distribution of the sample: Supplementary Fig. S3). Date of collection of specimens (first and last date) and median, minimum (earliest) and maximum (latest) dates of conception estimated from foetal age based on foetal length (see Methods). Indices of energetic state: carcass mass (kg), total amount of dissectible fat (kg) in carcasses, depth of subcutaneous fat over the rump (mm), muscle indices for *M. gluteobiceps* and *M. semitendinosus* and rumen dry matter (kg; see Methods). Values in braces exclude one outlier (specimen 12/1990; Fig. 5). ^a^ mean. *** *P* <0.001 (Table 4: models 2 and 6).

| Parameter | Year | Date of collection | Median | Min. | Max. | Range (days) | % within 4 days of the median date |
| --- | --- | --- | --- | --- | --- | --- | --- |
|  |  |  |  |  |  |  |  |
| Date of conception | 1988 | 21 – 28 Nov. | 19 Oct. | 13 Oct. | 26 Oct. | 14 | 72.2 |
|  | 1989 | 29 Nov. – 14 Dec. | 24 Oct. | 16 Oct. | 28 Oct. | 13 | 65.2 |
|  | 1990 | 23 Nov. – 8 Dec. | 26 {25} Oct. | 19 Oct. | 6 {2} Nov. | 19 {15} | 81.0 {85.0} |
|  |  |  | *** |  |  |  |  |
|  | All years | 21 Nov. – 14 Dec. | 22 Oct. | 13 Oct. | 6 {6} Nov. | 15.3 {14} ^a^ | 72.6 {73.8} |
|  |  |  |  |  |  |  |  |
|  |  |  |  |  |  | *n* |  |
| Carcass mass (kg) | 1988 |  | 35.8 | 29.5 | 45 | 18 |  |
|  | 1989 |  | 32.5 | 28.5 | 46 | 23 |  |
|  | 1990 |  | 33.2 {32.5} | 27.8 | 44.5 | 21 |  |
|  | All years |  | 34 | 27.8 | 46 | 62 |  |
|  |  |  |  |  |  |  |  |
| Total dissectible fat (kg) | 1988 |  | 14.5 | 6.9 | 18.1 | 18 |  |
|  | 1989 |  | 11 | 3.7 | 18.6 | 23 |  |
|  | 1990 |  | 11.5 {11.3} | 7.7 | 21.2 | 21 |  |
|  | All years |  | 12.6 | 3.7 | 21.2 | 62 |  |
|  |  |  |  |  |  |  |  |
| Rump fat depth (mm) | 1988 |  | 49 | 33 | 56 | 18 |  |
|  | 1989 |  | 42 | 26 | 58 | 23 |  |
|  | 1990 |  | 43 {42} | 35 | 63 | 21 |  |
|  | All years |  | 45 | 26 | 63 | 62 |  |
|  |  |  |  |  |  |  |  |
| Muscle Index _gluteobiceps_ | 1988 |  | 11.3 | 2.6 | 14.5 | 18 |  |
|  | 1989 |  | 13.1 | 4 | 16.2 | 23 |  |
|  | 1990 |  | 12.7 {12.8} | 4.3 | 17.6 | 21 |  |
|  | All years |  | 12.6 | 2.6 | 17.6 | 62 |  |
|  |  |  |  |  |  |  |  |
| Muscle Index _semitendinosus_ | 1988 |  | 3.1 | 1.7 | 4.7 | 18 |  |
|  | 1989 |  | 4.4 | 2 | 6.1 | 23 |  |
|  | 1990 |  | 4.4 | 1.1 | 5.6 | 21 |  |
|  | All years |  | 4.2 | 1.1 | 6.1 | 62 |  |
|  |  |  |  |  |  |  |  |
| Rumen dry matter (kg) | 1988 |  | 1 | 0.3 | 1.6 | 18 |  |
|  | 1989 |  | 1.5 | 1.1 | 2.1 | 23 |  |
|  | 1990 |  | 1.6 | 0.8 | 2 | 21 |  |
|  | All years |  | 1.4 | 3 | 2.1 | 62 |  |

**Table S2.** Coefficients, goodness of fit statistics and significance of linear regressions of estimated date of conception on indices of energetic state at breeding (predictor variables) in Svalbard reindeer aged 1-11 years shown in Fig. 3 and Fig. S3.

| Predictor variable | Reproductive state | Intercept | Slope | *r^2^* | *P* |  |
| --- | --- | --- | --- | --- | --- | --- |
|  |  |  |  |  |  |  |
| Carcass mass (kg) | Lactating | 311.95 (8.44) | – 0.48 (0.26) | 0.10 | >0.05 | NS |
|  | Non-lactating | 309.82 (6.77) | – 0.41 (0.18) | 0.18 | <0.05 |  |
| Total dissectible fat (kg) | Lactating | 301.86 (2.86) | – 0.52 (0.26) | 0.11 | >0.05 | NS |
|  | Non-lactating | 304.47 (5.04) | – 0.67 (0.33) | 0.15 | >0.05 | NS |
| Muscle Index_gluteobiceps_ | Lactating | 295.60(3.11) | 0.06 (0.25) | 0.002 | >0.05 | NS |
|  | Non-lactating | 293.54(3.49) | 0.07 (0.28) | 0.003 | >0.05 | NS |
| Rump fat depth (mm) | Lactating | 306.28 (5.12) | – 0.24 (0.12) | 0.11 | >0.05 | NS |
|  | Non-lactating | 310.27 (7.92) | – 0.32 (0.16) | 0.15 | >0.05 | NS |
| Rumen dry matter (kg) | Lactating | 291.17 (2.80) | + 3.40 (1.80) | 0.10 | >0.05 | NS |
|  | Non-lactating | 289.71 (3.13) | 3.85 (2.50) | 0.03 | >0.05 | NS |
| Muscle Index _semitendinosus_ | Lactating | 293.79 (2.81) | 0.62 (0.66) | 0.03 | >0.05 | NS |
|  | Non-lactating | 296.00 (3.61) | – 0.42 (0.89) | 0.01 | >0.05 | NS |

**FIGURE S1**

**
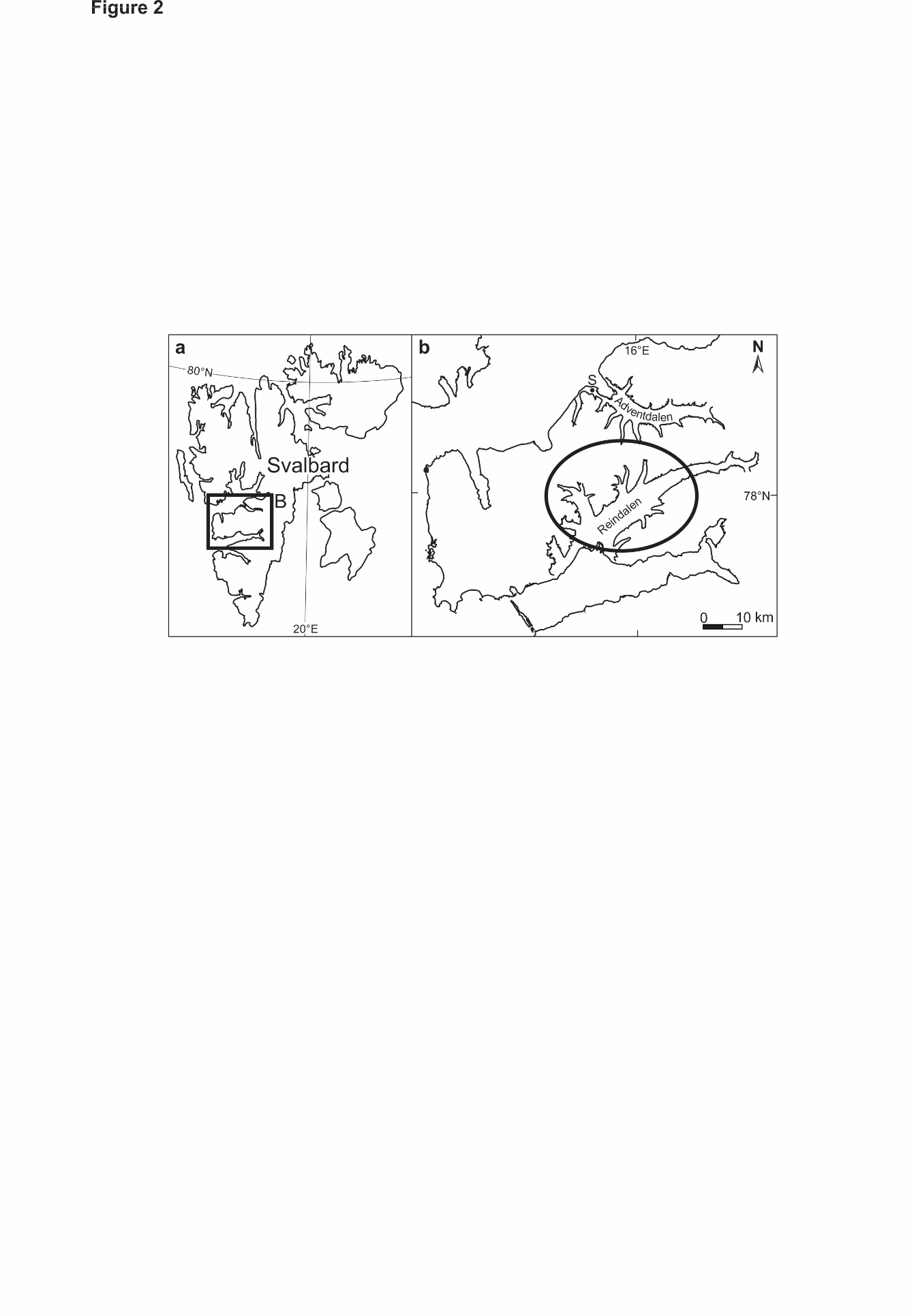
**

**Fig. S1**. (**a**) Map of Svalbard showing the location of the study area (box). (**b**) Study area delineated by the contour at 250 m above sea level. Date of calving (Fig. 1) was recorded in reindeer in Adventdalen and Reindalen. Date of conception was estimated in animals shot in the Reindalen-Semmeldalen-Colesdalen area (circled). S indicates Svalbard Airport where meteorological data (Fig. 1) were recorded.

**FIGURE S2**

**
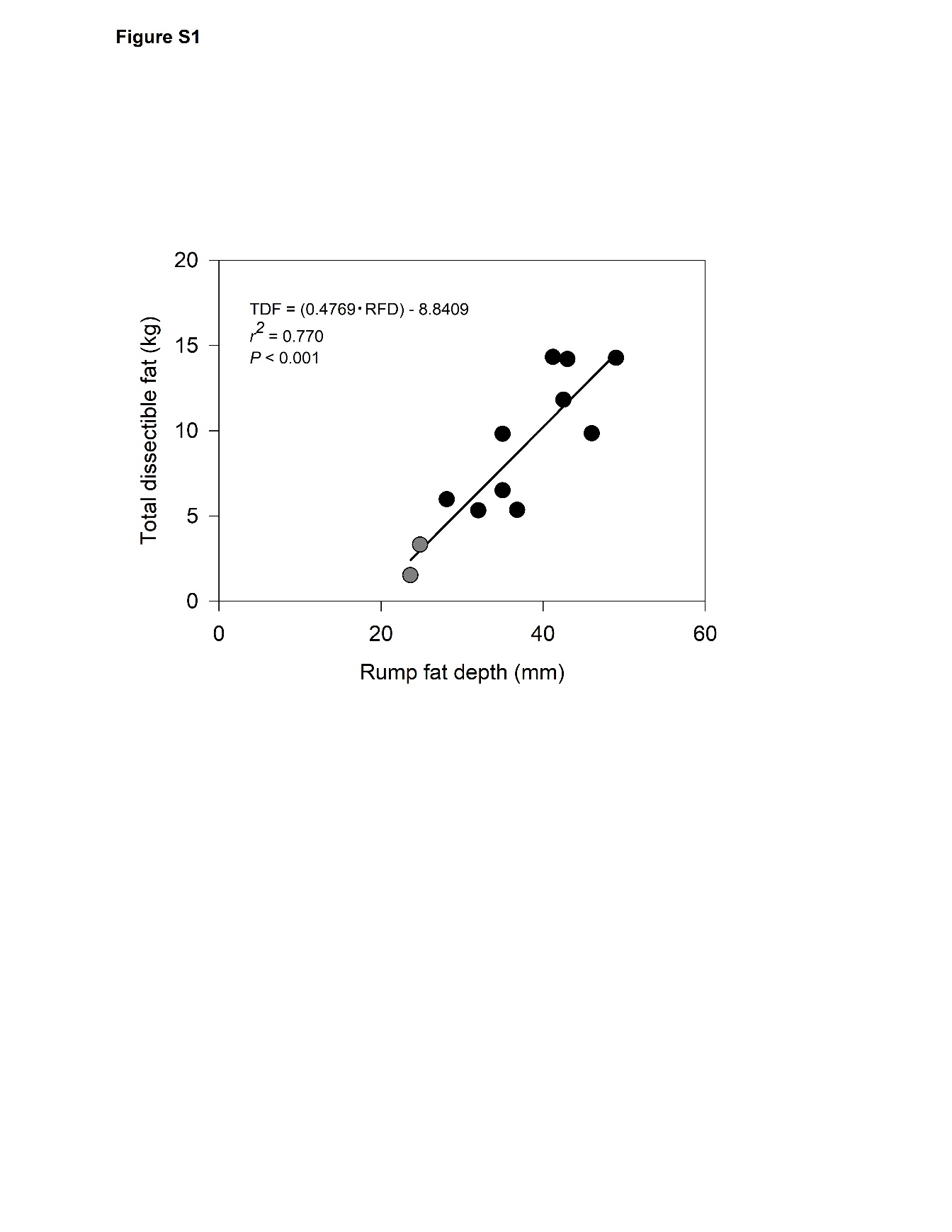
**

**Fig. S2.** Estimating energetic state. Relationship between the depth of subcutaneous fat over the rump (RFD, mm) and the total mass of dissectible fat in the carcasses (TDF, kg) in Svalbard reindeer. Data: females aged 2-11 yr. (mean 5.9 yr, s.d. 3.4, *n* = 10; black circles) and two calves (grey circles), all killed in late summer/early winter (range 14 August and 3 November; Tyler 1987). Linear regression: TDF fat (+ 1 s.e.m.) = -8.8409 (3.0711) + 0.4769 (0.0824) ∙ RFD, *r^2^* = 0.77, *P* <0.001).

Reference:

Tyler, N. J. C. Body composition and energy balance of pregnant and non-pregnant Svalbard reindeer during winter*. Symp. zool. Soc. Lond.* **57**, 203-229 (1987)

**FIGURE S3**

**
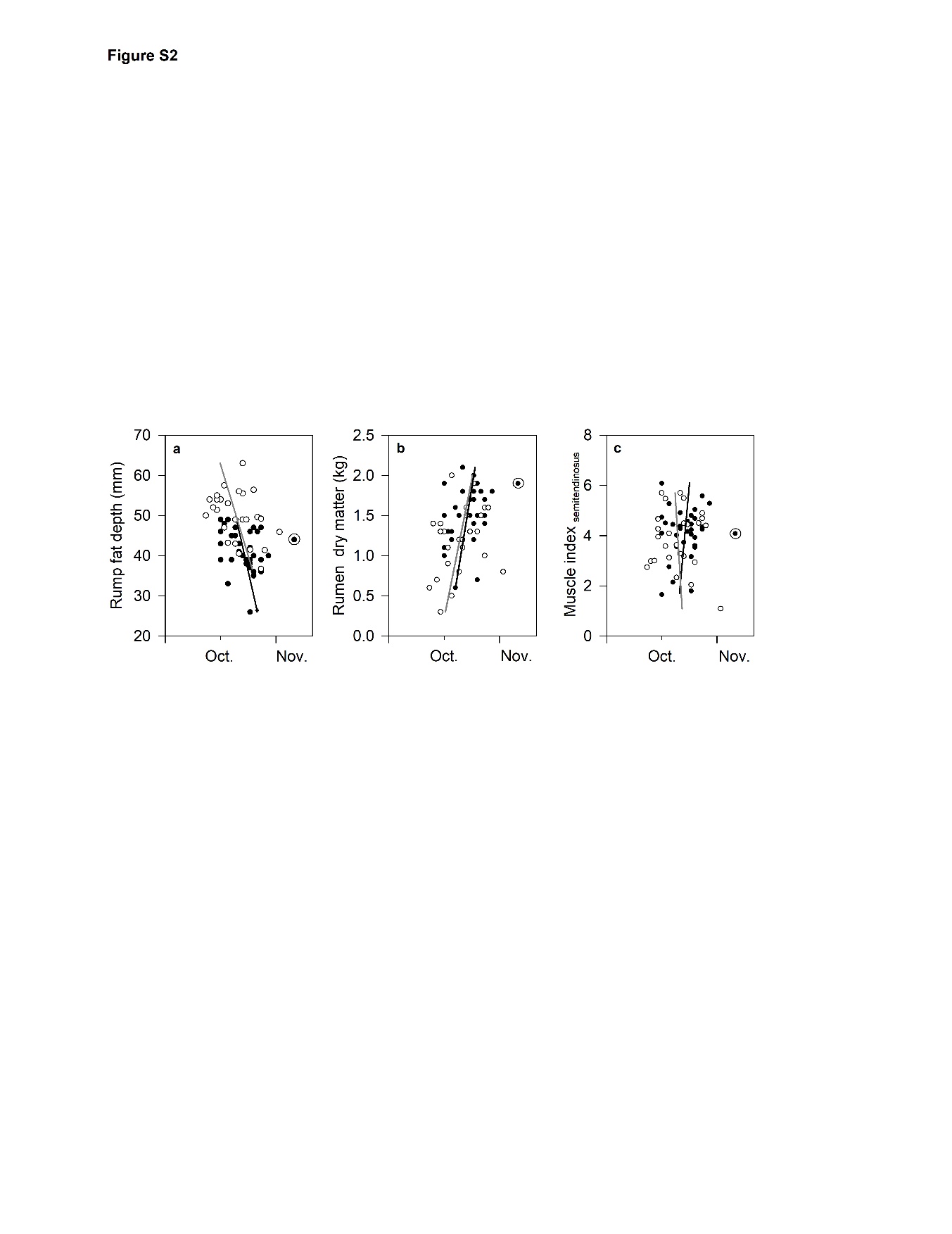
**

**Fig. S3**. Association between three indices of energetic state at breeding, additional to those shown in Fig. 3, on the date of conception (DoC) in Svalbard reindeer aged 1-11 years: (**a**) depth of subcutaneous fat over the rump (RFD, mm), (**b**) rumen dry matter (RDM, kg) and (**c**) Muscle Index*_semitendinosus_* (MI_s_; see Methods). Data are distinguished by reproductive state: lactating females (black circles, black regression lines); non-lactating (open circles, grey regression lines). By convention the axes are reversed so that the temporal variable is plotted on the abscissa. Coefficients, goodness of fit statistics and significance of linear regressions are given in Table S2. (Linear regressions for lactating females were calculated excluding one outlier (specimen 12/1990; ringed) identified on the basis of its standardized residual being >3.5 [Hopkins et al. 2009].) DoC was significantly associated with reproductive status singly and through interactions with age and RFD, respectively (Table 4) with age singly and through interactions with reproductive status, RFD and carcass mass, respectively (Table 4).

Reference:

Hopkins, W. G., Marshall, S. W., Batterham, A. M. & Hanin, J. Progressive Statistics for Studies in Sports Medicine and Exercise Science. *Med. Sci. Sports Exerc*. 41, 3–12 (2009).

**FIGURE S4**

**
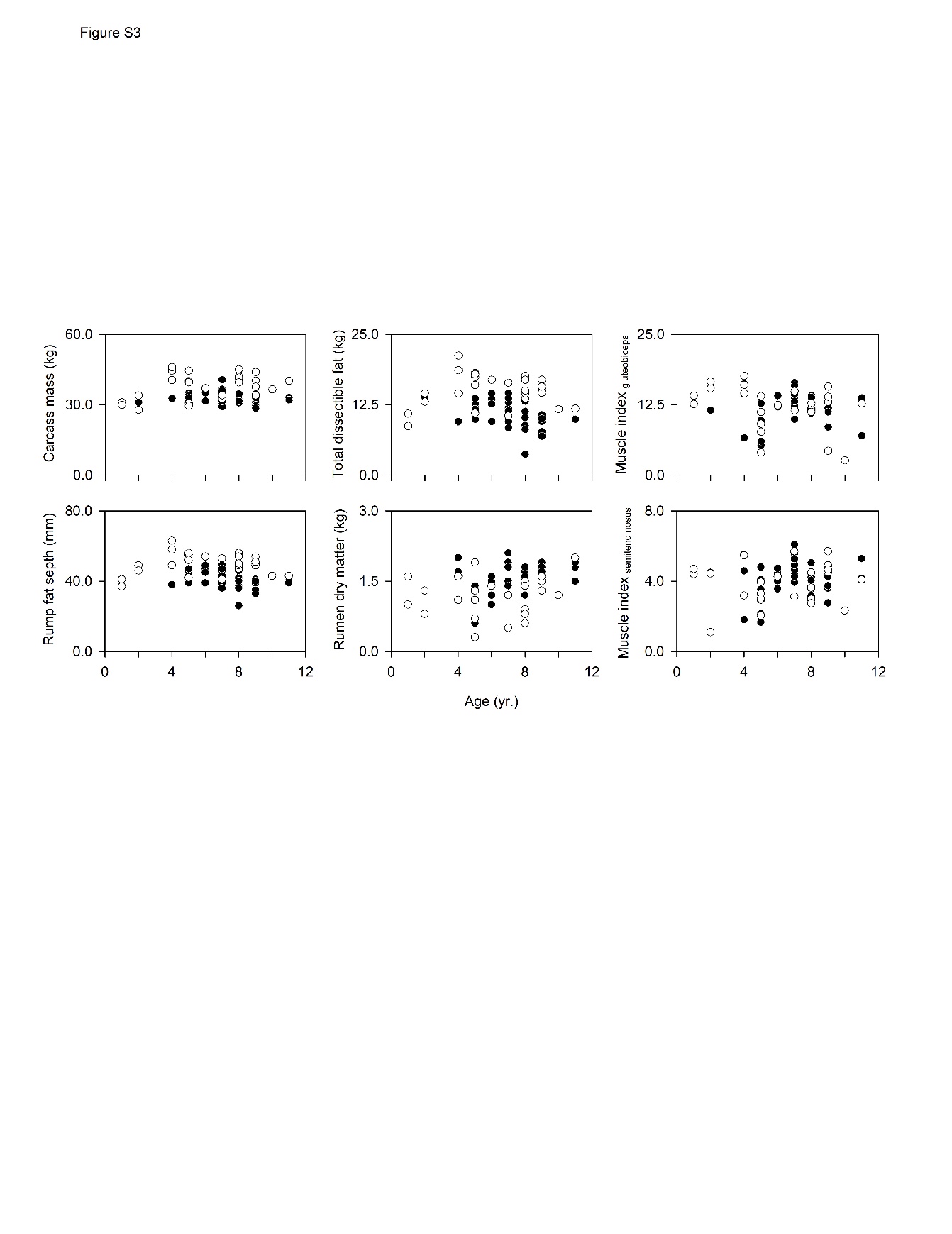
**

**Fig. S4.** Age-specific variation in six indices of energetic state approximately six weeks after mating in lactating (filled circles) and non-lactating (open circles) female Svalbard reindeer (*n* = 62): carcass mass (kg), total amount of dissectible fat in carcasses (kg), depth of subcutaneous fat over the rump (RFD, mm), muscle indices for *M. gluteobiceps* and *M. semitendinosus*, and rumen dry matter (kg; see Methods). Date of conception was significantly associated with age through interactions with carcass mass and RFD, respectively (Table 4: models 2 and 6).
